# Evolution's “Molecular Clock”: Not So Dependable After All?

**DOI:** 10.1371/journal.pbio.0020287

**Published:** 2004-08-17

DNA mutates, and it's a good thing it does. If it didn't, there could only be one kind of life, not the millions there are today, and species could not adapt to new challenges. This is because mutations in genes—the coding portion of DNA—are the raw material for evolution. However, genes make up a surprisingly small fraction of our DNA. If the genome were a cookbook, its 30,000-odd genetic recipes would be scattered among millions of pages of apparently meaningless nonsense.

Mutations affect all DNA, not just the genes, and this provides population geneticists with a veritable toolbox of methods useful, for example, in DNA profiling. Importantly, all these methods rely on the idea of a “molecular clock,” the notion that mutations rain down on noncoding DNA like a fine drizzle, so constantly that genetic similarity is a good measure of evolutionary time. Thus, if orangutans diverged from humans twice as long ago as did chimpanzees, on any given piece of DNA we would find twice as many differences between the orangutan sequence and the human sequence as between humans and chimps. The mutations are marking time.

If the molecular clock works, scientists can do wonderful things like estimating how long ago it was that the common ancestor of all humans lived, or when birds evolved from dinosaurs. The clock assumes that mutations occur independently of each other and at a constant rate. By analyzing thousands of noncoding DNA sequences scattered throughout the human genome, Edward Vowles and William Amos have found that the clock is anything but constant. Instead, a mutation in one spot in the genome affects the chance of getting another mutation nearby.[Fig pbio-0020287-g001]

**Figure pbio-0020287-g001:**
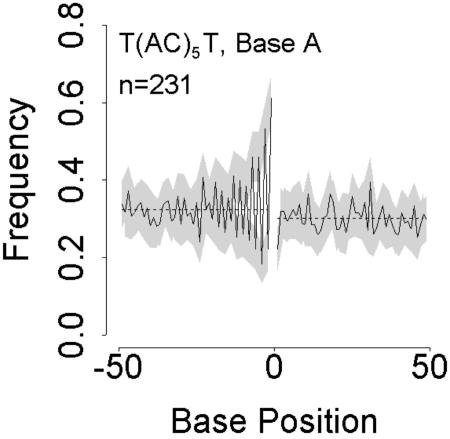
Non-random base frequencies around microsatellites

Not all noncoding DNA is made up of benign tracts of random letters. Some sequences appear to be more difficult to copy than others, and these trouble spots can give rise to alphabetic stuttering. DNA is made up of four component chemical units, called nucleotides, which are often referred to by their initial letters: A, C, G, and T. Stuttering occurs when the same pairs or triplets of letters occur together, for example ACACAC. Such regions are called microsatellites, and instead of mutating by swapping one letter for another, as most nucleotides do, these sequences evolve mainly by gaining and losing triplets or pairs like “AC.”

In this study, Vowles and Amos used the published sequence of the human genome to track down and compare thousands upon thousands of microsatellites. If the molecular clock ran smoothly, they would expect to find no similarity at all between the DNA sequences surrounding any pair of unrelated microsatellites. To their surprise, they found the complete reverse, with entirely unrelated microsatellites showing widespread and obvious similarities in their flanking DNA. This meant that mutations near microsatellites were not random, but favored certain letters in certain positions. Just as a new shipwreck will attract its own special community of marine life, so microsatellites appear gradually to change the surrounding DNA towards a common pattern. The result is convergent evolution, an unusual state of affairs where, as time goes by, DNA sequences become more similar, not less.

As yet, the exact mechanisms remain unclear, though it probably has something to do with how comfortably different combinations of letters sit next to each other. In English, “U” always follows “Q” and “B” never follows “V.” Similar rules may apply to DNA, albeit on a much subtler level. For example, if a microsatellite contains alternating As and Cs, the flanking regions also tend to have As at alternate positions, in phase with the As in the microsatellite. It is as if the DNA prefers the pattern in the microsatellite to extend into the flanking DNA, rather than abruptly stopping at the end of the microsatellite.

These findings suggest that it may be wise to take the notion of a molecular clock at face value. With a perfect clock, two or three identical mutations would be highly unlikely, but we now know that this may be possible near microsatellites. Vowles and Amos estimate that as much as 30% of the genome may show evidence of convergent evolution, simply because microsatellites are so common. These mutation biases probably exist to a lesser extent in most sequences. Once scientists understand more fully how and where these biases operate, they may be able to estimate more accurately the risk of any given mutation occurring, be it one that causes human disease or makes a virus more virulent. These findings represent yet another windfall from the Human Genome Project, and act as a powerful reminder that unexpected results always lurk around the corner as we delve deeper into the secret world of the genome.

